# The Effect of Size Statistics of the Background Texture on Perceived Target Size

**DOI:** 10.1038/s41598-018-29168-1

**Published:** 2018-07-19

**Authors:** Chia-Ching Wu, Chien-Chung Chen

**Affiliations:** 1 Department of Psychology, Fo Guang University, Yilan, Taiwan; 2 Department of Psychology, National Taiwan University, Taipei, Taiwan

## Abstract

We investigated the effect of the size distribution statistics of background elements on the perceived size of a target. We manipulated the first, second, and third order statistics (i.e., mean, variance, and skewness) of the background element size distribution. We used a two-interval forced-choice paradigm to measure perceived target size at different background size distributions. In each trial, a standard disk, or target, was presented in one interval with a textured background and a comparison disk, on a blank background, in the other. The task for the observers was to determine which interval contained a larger disk. We measured the point of subjective equality for the perceived target size with a staircase procedure. The perceived target size decreased with the increment of mean background disk size. The variance and skewness of the background element size did not affect the perceived target size. Our results showed that only the first order statistics of the background modulated the perceived target size, not the higher order statistics. A computational model, in which the visual system extracts size information by averaging the responses of different size channels, whose response is modulated by the size of the background elements, can account for the results.

## Introduction

The perceived size of an object depends not only on the size of its retinal projection but also its context. Many illusions demonstrate the effect of context on perceived target size, such as the Ebbinghaus illusion, the Ponzo illusion, the Müller-Lyer illusion, etc. In these illusions, observers may extract spatial or depth cues from the context and then use them to modify the perceived target size.

The Ebbinghaus illusion, or Titchener circle, is one of the best examples of the context effect on perceived size. In a typical Ebbinghaus illusion display, the perceived size of a central circular target is modulated by the size of the surrounding elements: a target circle surrounded by small circles appears larger than one surrounded by large circles. It is suggested that the Ebbinghaus illusion is a size-contrast effect^1–7^; that is, judgments of the size of the central circle are made in relation to the size of the surrounding circles. When the surrounding circles are small, the contrast mechanism causes an increment in the apparent size of the central circle; when the surrounding circles are large, the contrast mechanism causes a decrement in the apparent size of the central circle.

In a traditional Ebbinghaus display, all the surrounding elements are the same size and located at a fixed distance from the target. In daily life, however, an object is usually surrounded by other objects of different sizes and in different locations. Thus, the question is whether this effect of context on size perception can survive under such circumstances. One purpose of our study, then, is to investigate this issue. Furthermore, we investigate what factors may affect the illusion if the context effect does survive in this more natural scenario.

The natural environment, which may contains thousands of objects in a scene, is highly structured and predictable^8,9^. It is suggested that the visual system takes advantage of this and is able to grab the summary statistics in a natural scene to facilitate processing of the huge amounts of information contained in the environment^10–14^. One example is that people are remarkably accurate at computing averages, such as the mean size^15,16^, brightness^17^, orientation^15,18,19^, and location^20^, of a collection of objects. Hence, when an object is surrounded by many other objects of different sizes, our visual system may form an ensemble representation of the surrounding elements in various sizes, such as their first order statistics (i.e., mean), and use this summary statistics to make a size contrast.

The possible role of higher order statistics in context size effect is less clear. For the second order statistics, or the variance, in the summary statistics literature, it shows that an observer can compare size variance as efficient as mean^21^. However, there is also evidence showing that the variance of element size affects only the precision^22^ but not the mean of size estimation. Whether this  summary statistics property would affect context size effect remains an unresolved empirical question.

The third order statistics, or the skewness, is an index for the degree of asymmetry of the probability distribution. It is known that skewness plays an important role in material perception^23^ and texture discrimination (Chubb, Landy, & Econopouly)^24^. Thus, it can be interesting to test whether the skewness of the element size distribution of the surround stimulus, which is also a texture, could influence the perceive target size.

In this study, we used a background consisting of variously sized elements as the context stimulus and manipulated its size statistics to observe whether a change in the summary statistics affected the perceived size of the central target. In Experiment 1, we manipulated the first and second order statistics (i.e., mean and variance respectively) of the background size distribution to examine how a background with elements of different sizes affects perceived target size. Experiment 2 further investigated the effect of the third order statistics (i.e., the skewness) of the background size distribution. Note that in both experiments the background elements were randomly distributed to control the distance effect. We also used overlapping background elements filling the whole screen to control the density effect. We demonstrated that the perceived target size was modulated by the mean size of the background elements, but not their variance or skewness. We propose a computational model to account for the effect of context on perceived target size.

## General Method

### Ethics statement

This study was approved by the IRB of National Taiwan University Hospital (#201210026RIC, approval date: 27^th^ Nov 2012) and followed the guidelines of the Helsinki Declaration. Written informed consent was obtained from each participant.

### Apparatus

The stimuli were presented on a 24-inch LCD monitor controlled by a Macintosh computer via a Radeon 7200 graphics board which provided 10-bit digital-to-analog converter depth. The luminance and chromaticity of the monitor were measured with a PhotoResearch PR655 radiometer. The display had a mean luminance of 8.85 cd/m^2^ and mean chromaticity at (0.33, 0.33) in CIE 1931-xy coordinates. The refresh rate of the monitor was 60 Hz. The screen resolution was 1920(H) × 1200(V). The viewing distance was set such that each pixel made up 1′ of visual angle.

### Stimuli

In each trial, the stimuli included a test, which contained a standard disk on either a textured or uniform gray background, and a comparison, which was a disk on a uniform gray background. The display had a 32° visual angle extent. Both the standard and comparison disks had a black (luminance 0.22 cd/m^2^) and white (luminance 39.17 cd/m^2^) checkerboard pattern within their extents. The radius of the standard disk was 120 arcmin. The radius of the comparison disk varied across trials. The disks were placed at 4 ± *k* degrees visual angle to either the left or the right of the central fixation point, in which *k*, the jittering parameter, ranged from −0.08 to 0.08. The purpose of the position jittering was to prevent an observer from using local cues, such as the relative distance between the disks and the fixation, for the task. The background texture contained 5000 solid disks in various shades of gray, drawing from a uniform distribution, with luminance ranging from 1.05 to 26.98 cd/m^2^. They were randomly distributed across the whole display. The standard and comparison disks were fully visible, while background disks might be partially occluded by other disks.

### Procedures

We used a temporal two-interval forced choice (2IFC) procedure. Each trial contained two 500 ms stimulation intervals separated by a 600 ms inter-stimulus interval (ISI) and followed by a response period. In each trial, a test was randomly presented in one of the two intervals while the comparison was presented in the other. The standard and comparison disks were always presented on different halves of the screen so that the judgements would not be influenced by retinotopic aftereffects or looming/receding motion percepts. The task for the observers was to judge which interval contained a larger checkerboard disk. We used a one-up-one-down staircase method^25^ to determine the point of subjective equality (PSE). The initial radius of the comparison disk was randomly selected from either 83% to 92% (100 to 110 arcmin) or 108% to 117% (130 to 140 arcmin) of the test disk size. This randomization of initial comparison size was to avoid the effect of expectation. The step size was 1 arcmin, or the width of 1 pixel. The staircase procedure was terminated if the number of the trials was greater than 15 and the standard deviation of the comparison size in the last 7 trials was less than one step size, or 1 arcmin. The PSE estimate was the average of comparison size at the last seven trials. Each datum point reported was an average of four repeated measurements. Data from comparison disks at both the left and the right sides of the fixation were pooled together.

## Experiment 1

In Experiment 1, we investigated the effect of the first order (mean) and second order (variance) statistics of the background element size on the apparent target size.

### Method

**Figure 1:**
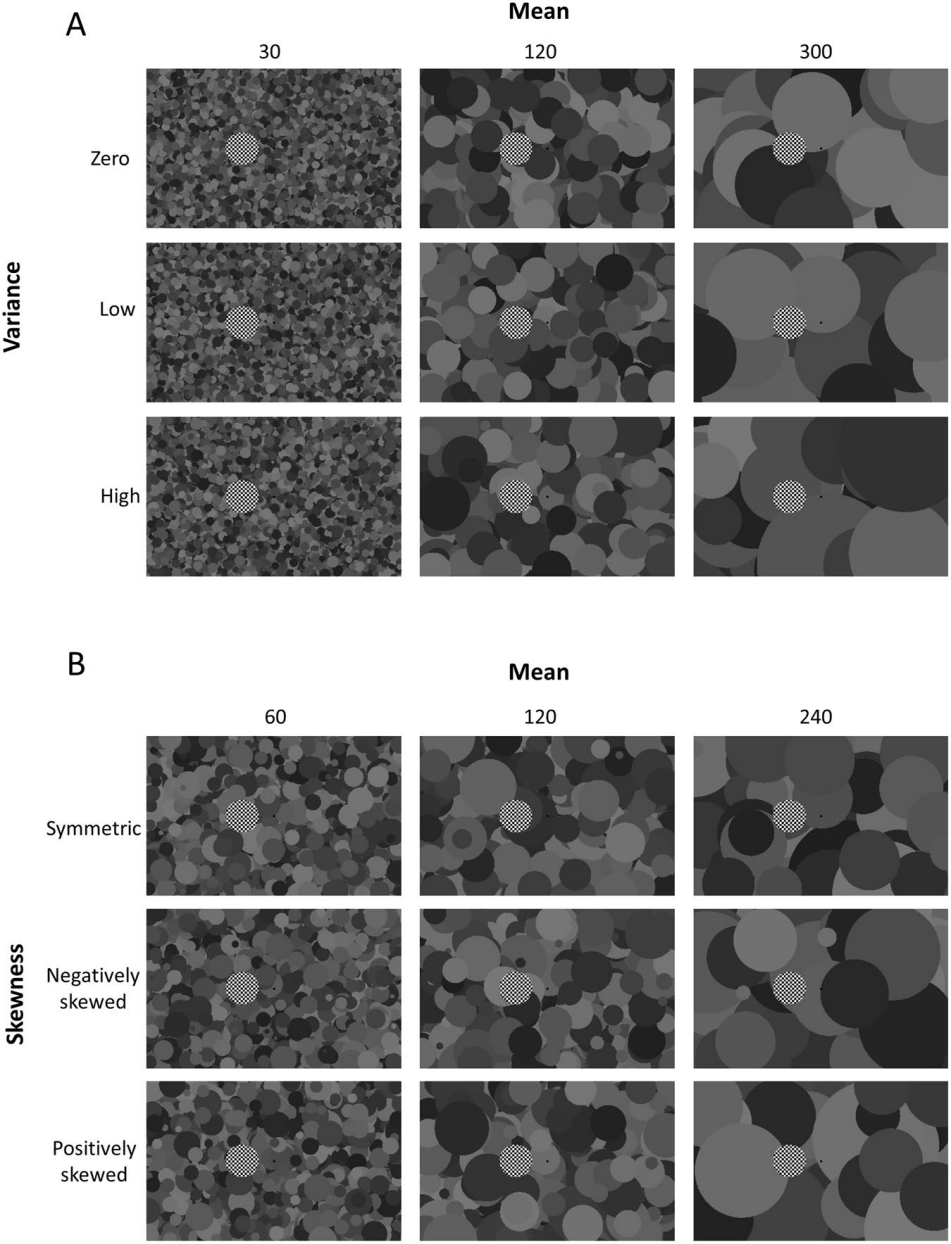
Example of the stimuli. (**A**) Example of the stimuli in Experiment 1. Each type of the stimulus has different size distribution with different mean and variance. (**B**) Example of the stimuli in Experiment 2. Each type of the stimulus has different size distribution with different mean and skewness. See text for the details.

In each trial, the radius of the background disks was drawn from the same Gaussian distribution (Fig. 1A). There were six possible values for the mean of the Gaussian distribution (30, 60, 120, 180, 240 or 300 arcmin). For each mean, there were three possible values for standard deviation: zero (all disks were the same size, subsequently called the zero VAR condition), 13% of the mean (the low VAR, condition), and 27% of the mean (the high VAR, condition). Thus, there were 18 different distributions in total. In addition, there was a baseline condition, involving a standard disk with a blank background. The sequence of conditions was randomized. Twelve observers participated in this experiment. The observers were naïve to the purpose of the experiment. All observers had corrected to normal (20/20) visual acuity.

### Results

**Figure 2:**
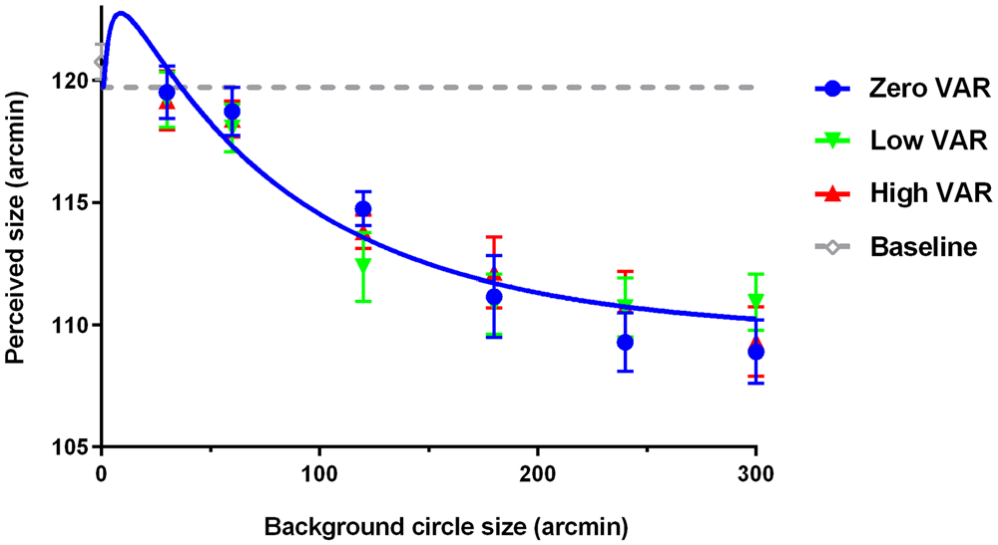
Perceived target size under background texture with different mean and variance combinations. The blue symbols stand for the data points of the zero variance condition, the green symbols for the low variance condition, the red symbols for the high variance condition, and the gray symbol for the baseline. The smooth curves are fits of the model. The gray dashed line represents the perceived target size in the baseline condition for comparison.

Figure 2 shows the average of the perceived target size, defined by the PSE of the reference size, measured with the background elements drawn from distributions with different mean and variance. The blue symbols stand for the data points of the zero VAR condition, the green symbols for the low VAR condition, the red symbols for the high VAR condition, and the gray symbol for the baseline. The smooth curves are fits of the model described below. For comparison, the perceived target size under the no-background condition was plotted as a gray dashed line. When there was no background texture, the perceived target size did not differ from the physical target size (*M* = 120.76, *SD* = 2.48, *t*(11) = 1.07, *p* = 0.31). When a background texture was presented, the perceived target size decreased with the increment of mean background disk size (Non-additive repeated measure ANOVA *F*(5,55) = 34.43, *p* < 0.001). The magnitude of the target size reduction was up to 9.25% of target size, similar to the Ebbinghaus illusion previously reported (around 3–15%)^4,7,26–29^. Note that the effect of the background texture was to decrease the apparent size of the target, even when the elements of the background texture were smaller than the target. This asymmetric effect, while counterintuitive, is well documented in the Ebbinghaus illusion literature^7,30,31^. Nevertheless, over all, the variance of the background element size distribution had no effect on perceived target size among the three (*F*(2,22) = 0.14, *p* = 0.87). There was also no interaction between the mean and variance of the background element size (*F*(10,110) = 1.35, *p* = 0.26).

Notice that, the effect of background element size statistics is mainly on the PSE. Neither the mean (additive repeated measure ANOVA *F*(5,187) = 1.38, *p* = 0.23) nor the variance (*F*(2,187) = 1.93, *p* = 0.15) had an effect on the slope of the psychometric functions. Since the slope affects the precision of the mean estimation, our result thus suggests that the measurement precision was not affected with background element size statistics.

Overall, our results showed that the perceived target size decreased with the mean size of the background disks, but not the variance. The variance did not interact with the mean to influence the perceived target size.

## Experiment 2

Experiment 2 further investigated how the human visual system represents and uses information on the skewness of the background element size. We manipulated combinations of the mean and skewness of the background element size and kept the variance constant.

### Method

In this experiment, we examined the effect of the third order statistics. To do this, we needed to find a way to modulate the third order statistics while keeping the first order statistics and the gamut of size modulation constant. For this purpose, we adapted an independent and identically distributed (IID) paradigm^32^ to determine the size distribution of the background texture (Fig. 1B). This paradigm provided a simple way to manipulate different moments of the distributions independently. The radius of the disks was drawn from a distribution whose probability density function (PDF) was determined by a linear combination of the first three order Legendre polynomials. Formally, let *L1*, *L2* and *L3* be the first three orders of Legendre polynomial, which is derived by applying Gram-Schmidt orthonormalization to the sequence of monomials, *x*, *x*^2^ and *x*^3^ in the range (−1, 1)^32^. The PDF of the distribution was defined as *f*(*x*) = *w0* + *w1* × *L1*(*x* − *x*_0_) + *w2* × *L2(x − x*_*0*_) + *w3* × (*x − x*_0_), where *x* was the radius parameter and *x*_0_ was the mean radius, which was either 60, 120, or 240 arcmin. The weighted parameters, *w0, w1*, *w2*, and *w3*, were selected in such a way as to make the skewness of the distribution either 0 (symmetric), −0.38 (negatively skewed), or 0.38 (positively skewed); the standard deviation of the disk radius was always 37% of the mean and the kurtosis was always 2.7. In addition, a condition in which a standard disk was placed on a blank background served as a baseline comparison. The sequence of conditions was randomized.

Twelve observers participated in this experiment. Among them, two observers also participated in Experiment 1. The observers were naïve to the purpose of the experiment. All observers had corrected to normal (20/20) visual acuity.

### Results

**Figure 3:**
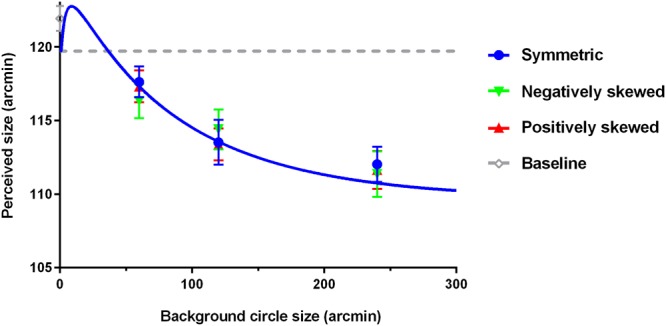
Perceived target size under background texture with different mean and skewness combinations. The blue symbols stand for the data points of the symmetric condition, the green symbols for the negatively skewed condition, the red symbols for the positively skewed condition, and the gray symbol for the baseline. The smooth curves are fits of the model and the gray dashed line shows the perceived target size in the baseline condition for comparison.

Figure 3 shows the effect of mean and skewness of the size distribution of the background elements. The blue symbols stand for the data points of the zero skewness condition, the green symbols for the negative skewness condition, the red symbols for the positive skewness condition, and the gray symbol for the baseline. The smooth curves are fits of the model described below and the gray dashed line shows the perceived target size in the baseline condition for comparison. However, in this set, the perceived target size when the background was blank differed slightly from the physical target size (*M* = 121.94, *SD* = 2.94, *t*(11) = 2.28, *p* < 0.05). When there was background texture, the perceived target size decreased with the mean size of the background circles, *F*(2,22) = 6.73, *p* < 0.05, similar to Experiment 1. The skewness of the background disk size distribution had no influence on the perceived target size, *F*(2,22) = 0.29, *p* = 0.75. Also, there was no interaction between the mean and the skewness of background circle size, *F*(4,44) = 1.18, *p* = 0.33.

The psychometric function slope was not affected by the background element statistics. Neither mean (*F*(2,88) = 2.38, *p* = 0.10) nor skewness (*F*(2,88) = 1.27, *p* = 0.29) showed an impact on the slope.

We also compared the perceived target size between the two experiments at mean background circle size 60, 120, 240, and a baseline condition. The results showed no difference between all the comparisons (all *t*(11) <1.97, *p* > 0.05).

Again, our results showed that the perceived target size decreased with the mean size of the background disks, similar to Experiment 1. The skewness, however, had no influence on the perceived target size when the variance was kept constant. Also, the skewness did not interact with the mean. Thus there was no joint effect of the mean and skewness on the perceived target size.

## Discussion

In this study, we investigated the effect of mean, variance, and skewness of the background texture size distribution on perceived target size. We measured the PSE of the target size for different combinations of mean, variance, and skewness of the background element size. Our results showed that the perceived target size decreased with the mean size of the background disks, but not with their variance or skewness. In addition, neither the variance nor the skewness interacted with the mean in producing an effect on perceived target size.

In our study, the inducers were the textured background with many randomly distributed elements in different sizes. This is different from the conventional Ebbinghaus display in which the inducers are all the same and arranged in an orderly fashion. Yet, our data showed a similar pattern to the conventional Ebbinghaus illusion, in which perceived target size decreases with the size of background elements. The magnitude of the size change, up to 9.25% of the target size, was also in the range of the conventional Ebbinghaus illusion (around 3–15%)^4,7,26–29^. This showed that the lateral effect on perceived size survives even when the inducing elements are randomly distributed in size and position.

Our result also shows that the perceived target size depended on the ensemble statistics of the background elements. This may suggest that the visual system is able to extract ensemble statistics of the background elements and use them to affect perceived target size. Furthermore, the perceived target size depends only on the mean size of the background elements, but not the variance or skewness of the size distribution. This suggests that the visual system mainly utilizes the ensemble statistics of the background element size to modulate target sizes by their mean, rather than their variance or skewness. That is, the extremely large or small background elements play little, if any, role in the size context effect.

### The Implication for Size Averaging

The above results also bring some important implications in size averaging. Recently, Ariely^15^ showed that observers can achieve great accuracy in judging the average size of an array of circles without knowing the sizes of individual items in the array. The mean size estimation is not affected by the number or density of the elements^15,22,33^, the variability of the element size^16,22^, the outliers^22,34^, the duration of presentation^16^, or even the spread of attention to other things^33,35,36^. The above is regarded as evidence that the size summary process is parallel and automatic^16,22,33,36,37^, although some argue that this process needs focused attention^38,39^. In our experiment, the observers were asked to make a size comparison between the standard and the comparison disks. The background texture received little, if any, attention. Hence, the context effect we measured here can be regarded as an implicit result of size averaging for the background elements. This implicit effect may be considered as the result of an automatic process for mean size computation and utilization without focused attention. In addition, the null effect of variance and skewness in our study also shows that the size averaging process is seldom influenced by the variability of the element size or the outliers, which is consistent with previous size averaging studies^16,22,34^. Hence, our study provides a valid implicit measure of size averaging.

Since there is an argument that an observer can process only a limited number of elements from the display for ensemble statistic calculation, it is reasonable to question whether such focal attention effect applies to our result. That is, the size context effect may depend on just a small number of background elements. An extreme case would be that in each trial an observer only focuses on just one background element, which in turn affects the perceived target size. Over many trials, the overall effect of those attended elements may be similar to the effect of mean statistics even though there is no ensemble statistics calculation in each trial. This issue can be resolved by observing the stability of the measurements. According to the central limit theorem, the variance of sample mean distribution decreases with the number of samples. Thus, in our high variance condition, an observer who samples only a handful of background elements should experience a greater trial-to-trial variation in mean background element size than one who samples many. If the sample size is large enough, the variance of the mean background element size may even be smaller than that of internal noise. As the result, the observer would experience a trial-to-trial variation not much greater than that in the no-variance condition, which is limited only by internal noise^40^. Therefore, if the perceived target is influenced only by a handful of background elements, compared with that in the no variance condition, the observer’s performance in the high variance condition should be more unstable and thus more trials were needed for the staircase procedure to converge. To test this hypothesis, we conducted a two-way ANOVA to analyze the number of trials used to reach stable PSE measurement in each condition of experiment 1. There was no main effect of background element size variance on the number of trials (*F*(2,22) = 2.19, *p* = 0.14). The averaged number of trials in the high variance condition (*M* = 22.36, *SD* = 2.27) was not significantly different from that in the low (*M* = 21.68, *SD* = 1.02) or zero variance (*M* = 23.05, *SD* = 2.47) conditions. This trend was consistent across conditions of different mean size, that is, no interaction observed (*F*(10,110) = 0.74, *p* = 0.57). Hence, the context effect observed in our experiment is likely resulted from a mechanism that samples a number of background elements large enough to reduce the trial-to-trial variability below measurable level.

Recently, Im and Chong^41^ had four Ebbinghaus sets, each with one target disk surrounded by 5 to 8 inducers, presented on both the left and right sides of the screen. The surrounding inducers on the left and right sides were of different sizes. The observers were to determine which side had the larger mean target size. They found that the observers perceived smaller mean size among targets with larger inducers. Hence, they concluded that the computation of the mean size was based on the perceived size rather than the physical size. If one postulates a sequential or a hierarchical processing model, one would conclude from Im and Chong^41^ that the Ebbinghaus illusion, and thus lateral size modulation, precedes mean size computation. On the other hand, our result, which shows that the perceived target size decreased with the increment of mean background element size, would imply that mean size computation precedes lateral size modulation. To resolve these two seemingly contradictory conclusions, one may consider a system in which lateral size interaction occurs at the element-to-element level everywhere on the display. When the task requires an observer to assess the size of a target embedded in the background, as in our experiment, the performance of an observer is influenced by lateral effects from every background element. Thus, it would seem the visual system pools the effects from all background elements first. Suppose that the task requires an observer to estimate the mean size of several targets, as in Im and Chong^41^. In this situation, the target size information available to the visual system is modulated by the surrounding elements. Thus, it would seem the surrounding modulation precedes size estimation.

### Mechanisms Underlying the Context Effect for Size

In the size perception literature, one popular account for the Ebbinghaus illusion is size contrast^1–7^. That is, larger surround elements make the central target appear small, while smaller ones make it seem large. Another explanation is the perspective theory^42^, which suggests that the size illusion is caused by the apparent distance implied by the size of the surrounding elements: that is, a larger surround implies a near context while a smaller surround implies a far context. Similarly, this theory would predict that larger surround elements would make the central target appear small and smaller ones would make it large. However, our results showed a unidirectional effect of size modulation. The presence of the background always decreased the apparent target size. It is just that this effect increased with background element size. This result is actually consistent with most Ebbinghaus effect measurements reported in the literature^7,30,31^, which show that the presence of inducers reduces apparent target size in most circumstances (except when the inducers are very small *and* very close to the target). Thus, it is clear that our effect cannot be explained by either size-contrast or implied depth theories. Below, we propose a model to account for this effect.

### Model

Even though some researchers suggest that there is no individual receptor in the early visual system that is specific to size, unlike other image properties such as motion direction or orientation^37^, there are many ways that a neural mechanism can encode size-specific information. For instance, the response of an end-stopping cell^43–46^ increases with the length of a stimulus in its preferred orientation up to a critical amount, and then decreases as the stimulus length further increases. This indicates that these end-stopping cells have a specific length tuning. There is also psychophysics evidence for this end-stopping mechanism at the behavior level^47,48^. Size information can also be extracted by spatial frequency channels^37,49–55^. The receptive field of a spatial frequency selective mechanism normally has distinct excitatory and inhibitory regions. The width of these regions determines the scale of the luminance modulation and in turn the spatial frequency selectivity of the mechanism. Thus, it is safe to assume that there are multiple mechanisms, each tuned to a particular size, in our visual system.

Let us assume that the tuning function of a size channel is a Gaussian function in log scale. Such tuning in log scale is known to other image dimensions such as spatial frequency^56^. The excitation of the *i*-th size channel (*E*_*i*_) to a target or a disk is proportional to its sensitivity or tuning function, *SE*_*i*_, a Gaussian function here. That is,

1 $${E}_{i}={w}_{i}\cdot S{E}_{i}={w}_{i}\cdot exp(-\frac{{(\mathrm{log}({S}_{t})-\mathrm{log}({S}_{{p}_{i}}))}^{2}}{2{{\sigma }_{i}}^{2}}),$$

where *S*_*t*_ represents the size of the target, *Sp*_*i*_, the peak size sensitivity of the *i*-th channel, *σ*_*i*_, the scale parameter, or the standard deviation, of the *i*-th channel’s tuning function, and *w*_*i*_, a scaler for the gain of the *i*-th channel. Hence, the more sensitive the channel *i* to the target, the larger excitation it has. Note that there can be an overlap between the tuning functions of different channels.

Each channel *i* receives inhibition from lateral channels with similar size tuning. Notice that, we empirically found that there is no effect of variance and skewness on target size. Thus, we can simply assume that the net inhibition received by the target channel depends only on the average of the effect of all background elements. That is, the visual system has extracted the mean size of the background elements first and uses that as the inhibition signal. As a result, the overall inhibition from the lateral channels to the *i*-th channel is expressed by

2 $${I}_{i}={v}_{i}\cdot {w}_{i}\cdot exp(-\frac{{(\mathrm{log}({\bar{S}}_{b})-\mathrm{log}({S}_{{p}_{i}}))}^{2}}{2{{\sigma }_{i}}^{2}})$$

where ${\bar{S}}_{b}$ represents the mean size of the background elements and *v*_*i*_, the weighting of the inhibition from the channels elsewhere. The response of the *i*-th channel is its excitation, *E*_*i*_, divided by a divisive inhibition term *I*_*i*_ plus an additive constant *z*. That is,

3 $${R}_{i}=\frac{{E}_{i}}{{I}_{i}+z}.$$

The perceived target size (*S*_*p*_) is estimated by averaging the responses of all the size channels weighted by their peak size. That is,

4 $$Sp=\frac{{\sum }^{}{S}_{{p}_{i}}\cdot Ri}{{\sum }^{}{R}_{i}}$$

Such an averaging operation is common in population coding literature. For instance, in the disparity averaging model, the human visual system calculates weighted responses of two separate spatial frequency mechanisms to estimate the perceived depth from disparity^57,58^.

#### Model implementation

To constrain the number of free parameters, we set the number of channels to be three, that is, small, medium, and large size channels. The peak sensitivity of the medium-size channel was 2.0791 log units, corresponding to the size of the standard disk. The peak sensitivity of the small- and large-size channels were set at *d*_1_ and *d*_2_ log unit below and above the medium-size channel respectively. The scale parameter, *σ*_*i*_, of the channel’s sensitivity function was half the peak of that channel.

Since there was no difference between the conditions of different variance and skewness, we set the parameters *w*_*i*_, *v*_*i*_, and *z* to be the same in these conditions to account for the relation between perceived target size and mean background element size. The scaler *w*_*i*_ for the three size channels was set at 1. In addition, we fixed the parameter *v*_*i*_ at 0 in the baseline condition since there was no background texture that could elicit and then modulate the perceived target size, and at 1 in the other three conditions to represent the context effect. Hence, with these constraints, our model contained 3 free parameters, *d*_1_ for the small-size channel, *d*_2_ for the large-size channel, and *z* for all channels.

#### Model performance

The model fit results show that the scalers *d*_*1*_ and *d*_*2*_ are 0.12 and 0.86 respectively. The model fits are shown as smooth curves in Figs 2 and 3. This model explains 93.23% of all variability in the perceived target size for the background textures. The root mean square error (RMSE) is 0.94 across observers, on par with the averaged standard error of the measurement.

Setting all the same parameters for different variance and skewness conditions did not reduce the goodness-of-fit of our model. This is consistent with the ANOVA (See Results) showing that there was no effect of variance or skewness on the perceived target size. The effect of the mean background element on the target was achieved through the tuning function of the size channels and the divisive inhibition processes. When there are no background elements, the target itself elicits excitation in a band of size channels. The visual system decides the perceived size of the target by averaging the size information across all elicited channels according to their responses (Eq. (4)). When the background elements are presented, they also produce an excitation in the corresponding size channels. Here, since there is no variance or skewness effect, we can safely assume that the effect of this excitation is represented by the mean size. Through a lateral interaction^59^, this excitation elicited by the background elements provides a divisive inhibition signal in the corresponding size channels at the target location. A change in the mean background element size will result in a different amount of inhibition according to the tuning function of the size channels, and therefore a different response reduction in each channel, resulting in different weighting of that channel in the channel response averaging process. For instance, a larger mean background element size would reduce the weighting of the larger size channel more than the others. Thus, the perceived target size would be smaller. The unidirectional result we got implies that the smallest size channel in the visual system is tuned to a size far smaller than the smallest stimuli we used. Thus, regardless which background was used, it always produced a greater response reduction in the larger channels than this smallest channel. As a result, the presence of the background reduced the perceived target size in the measurable range.

Notice that, when the mean background element size is very small and close to the peak of the smallest size channel, it is possible that the presence of the background can enlarge the perceived target size, shown as a bump at the left end of the plots in Figs 2 and 3. While this bump is beyond the range of our measurement, it is consistent with previous studies showing that a weak increase in the apparent target size is possible when the elements were less than 1/4 of the target size^7^.

### Alternative Interpretations

In a psychophysical study of visual illusion, it is always difficult to separate perceptual bias (i.e., illusion) from response bias (e.g., preference). Thus, some might argue that if an observer had a tendency to select the disk on a blank background over the one on a textured background as the larger one, then, the performance of this observer would be an underestimation of target size on textured background as our data showed. However, in our result, not only the perceived target size decreased with the background but also the magnitude of decrement increased with background element size. Response bias cannot explain the latter for it depended on a change of background element size, which is an image property. Thus, the effect must be perceptual.

There are context effects on perceptual dimensions other than size. There are many studies showing that the contrast threshold of a periodic target can be modulated by the presence of a context pattern with similar image properties such as orientation^60,61^, spatial frequency^60^, phase^62^, or color^63^. There are also reports showing that the perceived contrast of a central pattern can be modulated by the contrast of the context^64,65^. Thus, some might wonder whether the mechanisms underlying our effect may be similar to those underlying the contrast based context effects mentioned above. However, this class of context effects is unlikely to be a major cause of our result. First of all, many of the contrast based context effect depend on a particular spatial relationship between the target and the context. In our experiment, all background elements were randomly distributed across the whole display and were updated in each trial. Hence, there should be no systematic local structure effects. Second, many of those context effects vary with the contrast of the context. In our experiments, the mean and the range of luminance of each background texture was the same and the luminance of each background element was randomly selected from a uniform distribution. That is, there should be no systematically co-occurrence of luminance or contrast difference with size manipulation. Furthermore, the dependent variables of this class of context effect were either contrast threshold or perceived contrast. However, unlike brightness, which is known to affect perceived size^66,67^, there is no clear evidence showing that a change of perceived contrast or sensitivity could change it. For an empirical test of this issue, we rerun our experiment in two observers in the zero-variance condition with a target at the 50% of the original contrast level to simulate the change in perceived contrast or a reduction of contrast sensitivity. We found that the perceived size did not change with target contrast (*t*(3) = −0.39~2.07, *p* = 0.13~0.71). Hence, these in no evidence that the contrast based context effects play a role in our result.

Malkoc, Mulligan and Webster^68^ examined how the color distribution of a texture background affects the color salience of a target. They found that prior adaptation to the backgrounds enhanced target search on the same background. Some may argue that, in our experiments, successive presentation of the background texture with the same luminance distribution may lead to an adaptation to background luminance statistics and thus may make the target on the texture background more salient than the one on blank. However, notice that, being more salient does not necessary make the target bigger. There is known casual relation between the two. Furthermore, our result shows not only the perceived target size decreased with the background but also the perceived size decreased with the increase of background element size. Thus, even if there is a salience induced perceived size change that can account for the former effect, it cannot explain the difference in perceived size across different background texture conditions as the mean luminance and chromaticity of our textures was the same in all conditions. In addition, our standard disk with texture was randomly presented in one of the two intervals while the comparison on blank was presented in the other. Hence, even there is any adaptation effect, it should be applied to both standard and comparison disks and negate any adaptation effect. Hence, adaptation cannot account for our result.

## Conclusion

In sum, our results showed that the mean, but not variance or skewness, of the background size distribution influenced the perceived target size. The perceived target size decreased with the increment of the background element size. Our results cannot be explained by either the size-contrast theory or perspective theory. Instead, they can be explained by a model implementing a divisive inhibition and an averaging process over size channels.

## Data Availability

The datasets generated during and/or analyzed during the current study are available from the corresponding author on reasonable request.
